# Neoadjuvant Chemotherapy Creates Surgery Opportunities For Inoperable Locally Advanced Breast Cancer

**DOI:** 10.1038/srep44673

**Published:** 2017-03-22

**Authors:** Minghao Wang, Lingmi Hou, Maoshan Chen, Yan Zhou, Yueyang Liang, Shushu Wang, Jun Jiang, Yi Zhang

**Affiliations:** 1 Center of Breast Disease, Southwest Hospital, The Third Military Medical University, Chongqing, 400038, China; 2 Department of Thyroid Breast Surgery, The Affiliated Hospital of North Sichuan Medical College, Nanchong, Sichuan, 637000, China; 3 Department of Breast and Thyroid Surgery, Suining Central Hospital, #127 De-sheng Rd, Chuan-shan District, Suining, Sichuan province, 629000, China

## Abstract

Neoadjuvant chemotherapy (NAC), the systematic chemotherapy given to patients with locally advanced and inoperable breast caner, has been proven to be of great clinical values. Many scientific reports confirmed NAC could effectively eliminate sub-clinical disseminated lesions of tumor, and improve long-term and disease-free survival rate of patients with locally advanced breast cancer (LABC); however, up to now, LABC is still a serious clinical issue given improved screening and early diagnosis. This study, with main focus on inoperable LABC, investigated the values of NAC in converting inoperable LABC into operable status and assessed the prognosis. Sixty-one patients with inoperable LABC were initially treated with neoadjuvant chemotherapy; their local conditions were improved to operable status. Radical surgery was exerted on 49 patients. Original chemotherapy was performed after surgery, followed by local radiotherapy. And endocrine therapy was optional according to the hormone receptor status. The quality of life for most patients with skin diabrosis was obviously improved because their local conditions were under control. For all recruited cases, the survival duration and life quality were significantly improved in patients who finished both NAC and surgery compared to those who did not. Further more, this study demonstrates improved prognostic consequences.

Neoadjuvant therapy is the standard care for treatment of locally advanced and inoperable breast cancer. Application of this systematic therapy before surgery benefits patients with improved rates of breast-conserving surgery, increased possibility of early measurement of response, as well as potentially preferable outcomes for certain subgroups of high-risk patients. Although there is an exponential increase in the number of patients with earlier-stage operable breast cancers in the setting of improved screening and early diagnosis, locally advanced breast cancer (LABC) is still a severe clinical issue. Currently the definition of LABC is a widely used one without standard guidelines due to variations in LABC populations. Usually it is characterized by the following features: the diameter of primary lesion larger than 5 cm (T3), adhered and fixed skin to the chest wall (T4), fused regional axillary nodes (N2), and excluding remote metastasis^1^,^2^,^3^. LABC accounts for about 20% of breast cancer with 5 year overall survival rate ranging between 13 to 24% after simple operation, and local recurrence rate in 5 years is about 48%^4^. With the introduction of routine treatment methods such as radiotherapy and chemotherapy accompanying surgery for these patients, the 5 year survival rate now pegs at 30–55%^5^,^6^.

LABC can be classified into two types: operable and inoperable LABC. For the operable LABC, the typical therapeutic scheme is radical surgery combined with additional chemotherapy and radiotherapy. However, simply surgical resection may not be sufficient to excise the whole lesion in a case of chest wall adherence complicated with skin ulcer and edema. Previous attempts to treat such lesions were mostly characterized by early local recurrence and distance metastases, thus these cases were categorized into inoperable LABC^7^,^8^. And this type of inoperable LABC usually distinguishes from another inoperable subtype of breast cancer, the inflammatory breast cancer, which is featured with high degree of malignancy, no obvious mass and rapid progression of condition. In the past, simple radiotherapy or initial radiotherapy accompanied with surgery was conventionally chosen for treating inoperable LABC. Recently, many clinical trials have confirmed that neo-adjuvant chemotherapy could effectively eliminate sub-clinical disseminated lesions of tumor, and consequently improve the long-term and disease-free survival rate of patients with LABC. As a result, neoadjuvant chemotherapy combined with local therapy became a new treatment pattern of LABC^9^,^10^.

Therefore, the aim of the study was to assess the effectiveness of NAC for patients with inoperable LABC and to further evaluate the prognosis of the patients who could be potential beneficiaries of radical surgery and synthetic therapies due to the effectiveness of NAC.

## Patients and Methods

### Study population

A total of 1705 patients diagnosed with breast cancer were treated in our hospital from March 2004 to May 2009, among which 61 cases had primary inoperable LABC. The characteristics of these inoperable LABC included huge size tumors, ulceration, wide invasion or large inflammatory breast cancer, and no evidence of metastases. All patients were female, with ages ranging between 31 and 71 years old (average age: 55.1 years old). Pathology of theses cases indicated that 50 cases were infiltrating duct carcinoma, 5 cases of infiltrating lobular carcinoma, 2 cases of mucinous adenocarcinoma, 1 cases of squamous carcinoma, and 3 cases of medullary carcinoma. Finally, total 61 patients with primary inoperable LABC were enrolled for the present study.

### Study protocol

First of all, in order to obtain baseline characteristics of each patient, a complete physical examination and an interview were required to take by all the 61 patients. Clinical examination, mammography (MMG), ultrasound (US), and magnetic resonance imaging (MRI) were applied at the time of initial diagnosis to assess the status of primary tumor in the breast and lymph node. In addition, physical and special examinations were performed to exclude distant metastasis, such as bone scans and alkaline phosphatase detection were performed for detecting bone metastasis, chest X-ray and/or CT for examining lung transfer, liver ultrasound for liver metastasis and MRI for brain. After these most common organs were examined and excluded for distant metastasis, LABC was thus defined. If the patients situations were indicated, PET with 18-fluorodeoxyglucose (FDG-PET) or PET combined with computed tomography were performed prior to surgery. The diagnosis of primary breast cancer was established by core needle biopsies, and lymph node metastasis was confirmed by core needle biopsy or fine-needle aspiration or regional ablation at the ulcerated site. Then systemic chemotherapeutic tolerance of each patient was evaluated including blood cell count, liver function, ECG, chest X-ray or/and CT, bone scan, alkaline phosphatase detection and liver ultrasound. Of all the 61 cases, 49 cases acquired regional improvement after receiving 4 to 6 cycles of NAC and met the standards for radical surgery. The chemotherapeutic schemes included the methods of FEC (5-FU 600 mg/m^2+^ Epirubicin 90 mg/m^2+^ Cyclophosphamide 600 mg/m^2^) or TE (Paclitaxel 175 mg/m^2^ + Epirubicin 90 mg/m^2^). Prior to the third cycle of chemotherapy and surgery, US was utilized again to evaluate the size of tumor and LN of the affected breast. After completion of chemotherapy, MRI was performed once again to assess any residual disease. And what’s more, appropriate surgical treatments were applied to patients respectively according to their extents of residual tumor, followed by scheduled adjuvant radiotherapy. Due to poor therapeutic effect or some other reasons, there were 12 patients gave up the treatments after 1–4 cycles of NAC.

IHC staining for ER (clone 6F11, Novocastra Laboratories Ltd, UK), PR (clone 16, Novocastra) and Her2/neu (clone CB11, Novocastra) were carried out on formalin-fixed, paraffin-embedded tumor samples, prospectively in the context of the routine diagnostic service, using an automated staining system (Ventana Medical Systems, Inc.). Tissue sample expressing the antigen of interest were included on each histologic slide for positive control. IHC staining was evaluated by the local pathologists in accordance with national guidelines (www.sgpath.ch); in particular, with respect to the hormone receptors, the percentage of neoplastic cells involving in expression of either ER or PR was recorded, whereas Her2 expression was judged according to the guidelines of approved HercepTest by the Food and Drug Administration. Results were included in the histopathologic diagnostic report and communicated to the clinicians, as well as recorded in the dataset. Breast cancers with less than 5% of ER and PR expressions in neoplastic cells were considered as negative for hormone receptor expression. Weak or incomplete membrane staining (score 0 or 1+) was considered as Her2 negative, whereas strong and complete membrane staining (score 3+) indicated Her2 positive. There wasn’t any cancer with ambiguous expression of Her2 (score 2+) in the studied cases, all the them were either Her2 negative or Her2 positive. In this study, simple classification based on the expressions of ER, PR and Her2 was opted and advantageous since these three markers are routinely carried out in pathology laboratories, staining and evaluation protocols are well established worldwide and quality control programs are already available in several countries. We therefore classified breast cancer cases into four subtypes: luminal A (ER+and/orPR+, HER2−); luminal B (ER+and/or PR+, HER2+); BCL (ER−, PR−, HER2−) and Her2/neu (ER−, PR−, HER2+). Our clinical trial was successfully registered in the WHO International Clinical Trial Registry on May 29, 2014 with registration number ChiCTR-TRC-14005019. It was also approved by the Ethical committee of Southwest Hospital of the Third Military Medical University for carrying out this study with all the patients providing written informed consent. All experiments for this study were performed in accordance with relevant guidelines and regulations.

### Operative methods

After systemic operative tolerance assessment, there were 49 patients underwent appropriate surgery in two to three weeks after finishing NAC,. The operative methods included modified radical surgery for 13 cases, radical surgery for 27 cases and extended radical surgery for 9 cases. Here radical surgery was defined as removal of all breast tissue, including the nipple-areola complex, pectoris major and minor, axillary lymph nodes and the skin. Modified radical refers to removal of all breast tissue, the nipple-areola complex, skin and the level I and II axillary lymph nodes. Extended radical surgery means radical mastectomy and removal of internal mammary lymph nodes.

### Adjunctive therapy after surgery

All postoperative patients were resumed on 2 to 4 chemotherapies as per the original schemes. Then 4 weeks radiotherapies with dose of 45 Gy were exerted in the chest wall, supraclavicular fossa and medial breast region. Endocrine therapies were also performed if estrogen receptor (ER) or progestin receptor (PR) was positive with Tamoxifen for premenopausal women and aromatase inhibitors for menopausal women. Other chemotherapeutic schemes were chosen if the recurrence or metastases occurred.

### Statistical analysis

Survival rate analysis was performed using standard statistical computer software SPSS for windows (Version 13.0). Chi-squared test was used to determine the difference between groups, and the difference was considered statistically significant if P value < 0.05.

### Ethical approval and informed consent

The study was approved by the Ethical committee of Southwest Hospital of The Third Military Medical University and Institute and written informed consent was provided by the patients. All experiments were performed in accordance with relevant guidelines and regulations.

## Results

### Local controlling status after neo-adjuvant chemotherapy

**Figure 1:**
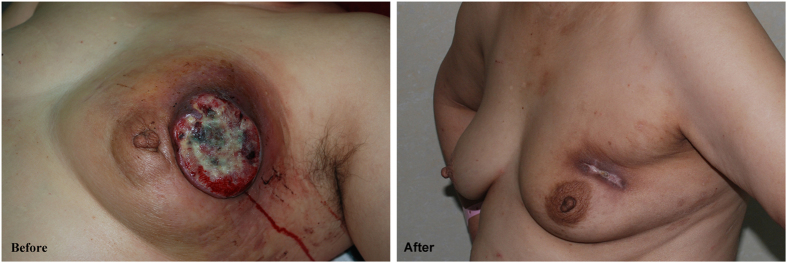
Before: Before chemotherapy, the tumor was complicated with regional ulceration and infection, and surgery was difficult. After: Ulcer healed and edema subsided after 5 cycles of primary FEC chemotherapy.

**Figure 2:**
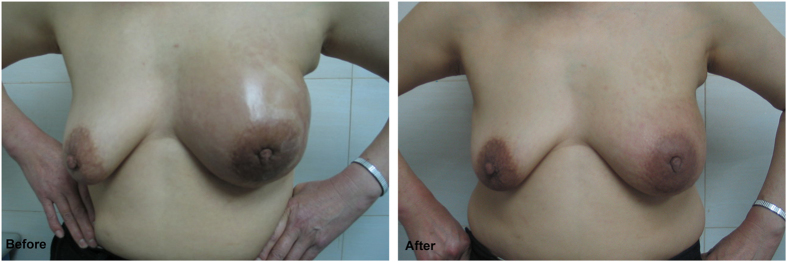
Before: Inflammatory breast cancer. The edema of left breast and surrounding skin were obvious, which made radical surgery impossible. After: The edema of breast subsided after 6 cycles of primary TE chemotherapy, which met the radical surgery standard.

The baseline clinical characteristics of the study population are shown in Table 1. A Total of 61 patients with inoperable LABC were identified. Twelve patients were exempted from surgery due to failure in completing the NAC cycles. Eventually there were 49 patients met the standards of radical surgeries with improved conditions including cytoreduction of tumors, henosis or remarkable decrease of ulcers, relief of adhesion between tumor and chest wall and extinction of breast edema, which helps to suture skin incision or graft small skin in radical surgery after successfully completing NAC, Figs 1 and 2. Based on the relations between tumors and muscles of chest wall as well as the metastases of local lymph node, 13 patients underwent modified radical surgery, 27 patients received radical surgery and 9 patients had extended radical surgery respectively.

### Local recurrence

Post operation follow-ups were conducted at least once every half year with follow up periods ranging between 1 to 6 years. There were 8 cases were followed up for more than 5 years, 19 cases more than 4 years, 29 cases more than 3 years, 42 cases more than 2 years and 49 cases more than 1 year. Eighteen cases suffered local recurrence on the skin of chest wall, inner mammary region or supraclavicular lymph nodes. Recurrence occurred in 4 cases within 1 year, 12 cases between 1 to 3 years, 1 case between 3 to 5 years, and 1 case after 5 years (Table 2).

### Distance metastases

Survival statistics of the patients who underwent surgery are shown in Table 1. Only the first metastatic sites were considered and analyzed. Contralateral breast or lymph node metastases were found in 4 cases, bone metastases in 12 cases, liver metastases in 7 cases and lung metastases in 5 cases. Metastasis occurred simultaneously in the contralateral site combined with bone in 2 cases, 2 cases in the contralateral site and liver and one case in liver and bone. 6 cases (6/49) of metastases occurred within 1 year, 20 cases (20/29) within 3 years (Table 2).

### Survival rate

**Figure 3:**
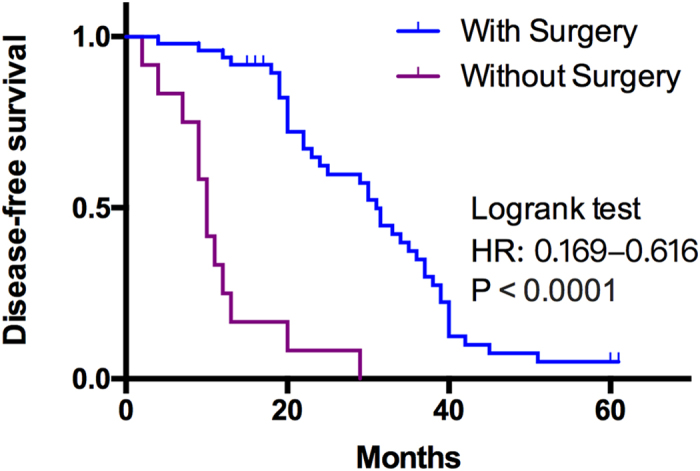
Survival curves according to surgery and without surgery.

After 1 to 6 years’ follow-up, 10 out of the 12 patients who did not undergo surgery died within 1 year and the other 2 patients died in the subsequent year. Among the 49 patients who received surgery, 32 cases are still living, 2 cases survived longer than 5 years (2/8), and 13 cases survived longer than 3 years (13/29) (Table 2). For the Disease-free survival (DFS), 1 case survived for more than 5 years (1/8) and 5 cases survived for more than 3 years (5/29) (Table 3). Survival curve indicated a better DFS in patients who had surgery than in those who hadn’t (HR, 0.619–0.616, P < 0.0001) Fig. 3. It shows from survival analysis that the survival durations of patients received synthetic treatments were significantly longer than those who didn’t (P < 0.01).

### Quality of Life

Obviously, patients with inoperable LABC acquired satisfactory improvements of local status and became eligible candidates for radical surgery through NAC. After surgical excisions of local tumors, ulcer and unbearable smell complications were eliminated although local recurrence rate and distance metastases rate were still relatively high. However, the patients could eventually resume their normal life with drastically improved living qualities compared with their living qualities before surgery. What’s more, ulceration relapsed in only one case before death of all the cases followed.

## Discussion

LABC principally represents primary breast cancer, it is classified into stages of T3, T4 and TXN2, excluding distant metastases and accounting for about 10 to 15% of all newly diagnosed breast cancers^1^. Local recurrence rate for LABC are usually high with an alarming low long-term survival rate. Haagensen and Stout had classified LABC into operable and inoperable types^11^. For operable LABC, radical surgery is routine intervention accompanied with post-operative chemotherapy and radiotherapy, and pre-operative NAC is rather optional for breast conservation purposes. But for inoperable LABC complicated with skin ulcer, skin edema and adhesion with chest wall, direct operation mostly failed to prevent early local recurrence and distance metastases. Consequently, effective treatments are employed to relieve infiltration before surgery for inoperable LABC. The preoperative treatment includes systematic chemotherapy and local radiotherapy. Previous studies have confirmed that LABC is usually complicated with distant micro-metastases, which could possibly develop into new metastases if only simple regional treatment measures were taken. Therefore, recent treatment schemes for LABC are usually centered on pre-operative NAC to control the local symptoms, which could be improved converting inoperable LABC into operable state. It has been proven that this method can effectively inhibit distance metastases and improve overall survival rate^12^,^13^.

Most clinical researches tailored Anthracycline as the principal agent of NAC scheme, and appended Taxane to improve the therapeutic effect of the chemotherapy^14^,^15^. Results from such studies showed that, about 3 to 46% of operable LABC cases could achieve pathological complete response (pCR) after NAC, and 80% could achieve partial response (PR)^16^,^17^. However, previous reports on the effectiveness of NAC on inoperable LABC were rather less promising. In the 61 cases of this study, there were 48 cases achieved PR, and only 1 case of inflammatory breast cancer resulted with pCR. Possible reasons for these results may because of severe local symptoms or insufficient chemotherapeutic cycles making it difficult achieve pCR by chemotherapy. This hypothesis notwithstanding, warrants further verification by clinical researches in the future.

It is showed from our findings that effective NAC could provide patients of inoperable LABC with operation opportunities. As discussed before, the local recurrence rate within 1 year was 8.16% (4/49), and 55.2% (16/29) within 3 years. What’s more, it shows the trend of declination in 3 years and peaks between 1 to 3 years. The low recurrence rate within 1 year might be attributed to the reasons such as follow-up effects of NAC, adjuvant radiotherapy and endocrine therapy after surgery. After 3 years, most patients died due to distant metastases, which might account for the decreased local recurrence. Comparing with local recurrence rate of LABC, it was 48% within 5 years as reported in previous studies^4^. For operable LABC with tumor larger than 10 cm in size, the recurrence rate was about 50% within 2 years after treating with synthetic therapies (surgery combined with chemotherapy and radiotherapy)^18^. However, recurrence rate of inoperable LABC was much higher due to severe infiltrations.

It is known that the most common parts for distant metastases of LABC including bone, liver, lung, brain, contralateral breast region and local lymph. As for inoperable LABC, distant metastases most often than not may occur without timely intervention although it is usually absent during clinical examinations^19^. Nearly all cases with poor therapeutic effects died as a result of distance metastases within 1 year in this study. The most common sites of primary metastases are the bone, contralateral breast and local lymph nodes, liver, lung and brain. And there also found simultaneous metastases in multi-organs. It is estimated that the causes for high rate of contralateral metastases were severe local infiltration making tumor cells easily invade into the infiltrated skin lymphatic network and internal mammary node, and eventually reach the contralateral normal region. In this study, distant metastases rate was about 12.2% (6/49) within 1 year, 69% (20/29) within 3 years, and 75% (6/8) within 5 years. Most of the patients who didn’t undergo surgery had distant metastases occurring within 1 year at a relatively higher rate compared to those patients who did undergo surgery.

In addition, inoperable LABC is featured with more severe symptoms and advanced classification compared with operable LABC, and subsequently causing higher mortality. The mortality rate after surgery in this study was 4.08% (2/49) in 1 year, 48.2% (14/29) in 3 years, and 75% (6/8) in 5 years. Although the mortality rate after surgery was higher than the total breast cancer population, it was significantly lower than the rates of patients without surgery, which are about 83.3% (10/12) in 1 year and 100% in 2 years. The results indicated that NAC combined with surgery could improve the survivals of inoperable LABC.

At last, the life quality of the inoperable LABC patients was dramatically improved after the treatments of effective NAC and surgery. Most patients had lived in great miseries and pain due to the complications of LABC, which includes foul ulcer, regional pains and obvious limb swelling. Therefore it is promising to note that severe ulcers for most cases were healed with no recurrence after effective NAC and surgery, and ulceration relapsed in only one case. And most patients with local recurrence were simultaneously accompanied with distant metastases in some critical organs, becoming the main lethal factors in LABC, thus the regional symptoms usually had not become worse before death. The patients couldn’t benefit with prolonged survival duration, fortunately the qualities of their daily life could be improved by appropriately controll of local symptoms.

In summary, inoperable LABC can be improved by NAC converting it into operable status. Although the long-term survival rate of these patients didn’t show obvious increase after systematic and synthetic treatment, the mean survival time and quality of life were considerably improved. As this study is by no mean perfect with short research time duration, limited number of follow-up cases longer than 5 years and incomplete clinical data, however, our findings proved that effective NAC could convert inoperable LABC into operable states, and significantly improve the prognosis. With better future follow-ups and a more comprehensive clinical data, the results of the synthetic treatment of inoperable LABC would be more convincing.

### Limitations

Nevertheless, some limitations worth mentioning in this study are the facts that only 61 cases were included and short follow up time varying between 1 and 6 years. The results would be more persuasive if we could include more cases and increase the follow-up time. Still, LABC is a major clinical problem in China and a common presentation in many parts of the world. In this study, it is proved that inoperable LABC can be converted into operable states by effective NAC, and prognosis be improved as well. Despite all of these defects, this article provides strong clinical evidence for LABC treatment.

## Conclusion

The present study demonstrates that effective NAC can convert inoperable LABC into operable states, and further improve the prognosis.

## Additional Information

**How to cite this article:** Wang, M. *et al*. Neoadjuvant Chemotherapy Creates Surgery Opportunities For Inoperable Locally Advanced Breast Cancer. *Sci. Rep.* **7**, 44673; doi: 10.1038/srep44673 (2017).

**Publisher's note:** Springer Nature remains neutral with regard to jurisdictional claims in published maps and institutional affiliations.

## Figures and Tables

**Table 1:** Clinical characteristics and treatment information of patients.

	Cases	%
Patients	61	100
Age (year)		
<50	23	37.7
>50	38	62.3
Clinical tumor stage		
T2	9	14.8
T3	36	59.0
T4	16	26.2
Clinical nodal status		
Negative	7	11.5
Positive	54	88.5
Grade		
IIb	29	47.5
III	32	52.5
Pathological type		
infiltrating duct carcinoma	50	82
infiltrating lobular carcinoma	5	8.2
mucinous adenocarcinoma	2	3.3
squamous carcinoma	1	1.6
medullary carcinoma	3	4.9
HR status		
Positive	29	47.5
Negative	32	52.5
HER2 status		
Positive	19	31.1
Negative	42	68.9
Breast cancer subtypes		
HR+/HER2−	15	24.6
HR+/HER2+	14	23.0
HR−/HER2+	5	8.2
TNBC	27	44.3
Chemotherapeutic scheme		
FEC	17	29.9
TE	44	61.1
Operative method		
No surgery	12	19.7
Modified radical surgery	13	21.3
radical surgery	27	44.3
Extended radical surgery	9	14.7

ER, estrogen receptor; PR, progestin receptor; HR, hormone receptor; HER2, human epidermal growth factor receptor 2; TNBC, triple negative breast cancer; FEC (5-FU 600 mg/m^2+^ Epirubicin90 mg/m^2^+ Cyclophosphamide600 mg/m2). TE (Paclitaxel 175 mg/m^2^+ Epirubicin 90 mg/m^2^).

**Table 2:** Survival statistics of patients with surgery and in different breast cancer subtypes.

Subtypes	>1 year (n = 49)	>3 years (n = 29)	>5 years (n = 8)
Survival without LR	45(91.8%)	13(44.8%)	1(12.5%)
Breast cancer subtypes			
HR+/HER2−	11(22.4%)	4(13.8%)	1(12.5%)
HR+/HER2+	12(24.5%)	3(10.3%)	0
HR−/HER2+	3(6.1%)	1(3.4%)	0
TNBC	19(38.8%)	5(17.2%)	0
Survival without DM	43(87.8%)	9(31.0%)	2(25.0%)
Breast cancer subtypes			
HR+/HER2−	11(22.4%)	4(13.8%)	1(12.5%)
HR+/HER2+	12(24.5%)	2(6.9%)	1(12.5%)
HR−/HER2+	2(4.1%)	0	0
TNBC	18(36.7%)	3(10.3%)	0

LR, local recurrence; DM, distance metastases.

**Table 3:** Survival statistics of inoperable LABC.

Treatment	Cases	>1 year	>3 years	>5 years	P
Without surgery	12	2	0	0	<0.01
With surgery	49	47/49	13/29	2/8	
